# Supplementary material for: The relationship of within-individual and between-individual variation in mental health with bodyweight: An exploratory longitudinal study
Source: PLoS One. 2024 Jan 10;19(1):e0295117. doi: 10.1371/journal.pone.0295117 (PMC10781195; doi:10.1371/journal.pone.0295117)
Supplement: S1 Table — PSS = Perceived Stress Score, PHQ = Patient Health Questionnaire, GAD = Generalised Anxiety Disorder questionnaire. (DOCX) [file pone.0295117.s001.docx]

***Table S1. Mean weight and mean mental health scores per month over the study period*** *(August 2020 – April 2021). PSS = Perceived Stress Score, PHQ = Patient Health Questionnaire, GAD = Generalised Anxiety Disorder questionnaire.*

|  | **Aug 2020** | **Sep 2020** | **Oct 2020** | **Nov 2020** | **Dec 2020** | **Jan 2021** | **Feb 2021** | **Mar 2021** | **Apr 2021** |
| --- | --- | --- | --- | --- | --- | --- | --- | --- | --- |
|  | **M (SD), n** | | | | | | | | |
| **Weight** | 77.15 (16)  n=1135 | 77.41 (15.77)  n=1328 | 76.8 (15.43)  n=1412 | 76.68 (15.31)  n=1319 | 76.65 (15.3)  n=1237 | 77.21 (15.75)  n=1254 | 76.89  (15.47)  n=1099 | 77.32  (15.69)  n=1153 | 77.02  (16.01)  n=1147 |
| **Stress (PSS-10)** | 10.95(6.62), n=442 | 10.81(6.7), n=912 | 11.03(6.94), n=1014 | 10.88(7.3), n=843 | 11.49(7.33), n=883 | 11.53(7.26), n=1307 | 10.96(7.45), n=1598 | 10.74(7.64), n=1585 | 10.23(7.72), n=1486 |
| **Depressive symptoms (PHQ-8)** | 3(3.5), n=456 | 2.98(3.5), n=952 | 3.12(3.56), n=1025 | 3.28(3.8), n=850 | 3.4(3.76), n=917 | 3.63(4.05), n=1301 | 3.46(4), n=1591 | 3.27(3.97), n=1581 | 3.09(3.93), n=1478 |
| **Anxiety symptoms (GAD-7)** | 2.55(3.38), n=470 | 2.58(3.2), n=1038 | 2.79(3.52), n=1109 | 2.73(3.48), n=920 | 2.82(3.41), n=971 | 2.97(3.68), n=1327 | 2.82(3.69), n=1598 | 2.74(3.68), n=1579 | 2.52(3.48), n=1484 |
