# Supplementary material for: The relationship of within-individual and between-individual variation in mental health with bodyweight: An exploratory longitudinal study
Source: PLoS One. 2024 Jan 10;19(1):e0295117. doi: 10.1371/journal.pone.0295117 (PMC10781195; doi:10.1371/journal.pone.0295117)
Supplement: S2 Table — Number and percentage (out of those who downloaded and onboarded to the app) of participants providing data per month per variable (stress, depression, anxiety, weight). Note: Participants were only asked to begin completing mental health questionnaires ~30 days after onboarding; therefore completion rates in August are low compared to subsequent months. (DOCX) [file pone.0295117.s002.docx]

***Table S2.*** ***Outcome completion rates***. *Number and percentage (out of those who downloaded and onboarded to the app) of participants providing data per month per variable (stress, depression, anxiety, weight). Note: Participants were only asked to begin completing mental health questionnaires ~30 days after onboarding; therefore completion rates in August are low compared to subsequent months.*

| **Month** | **Number that downloaded and onboarded to the app** | **Number that provided data on stress (%)** | **Number that provided data on depression (%)** | **Number that provided data on anxiety (%)** | **Number that provided data on weight (%)** |
| --- | --- | --- | --- | --- | --- |
| **August** | 1517 | 438 (28.9) | 452 (29.8) | 466 (30.7) | 1131 (74.6) |
| **September** | 2060 | 912 (44.3) | 952 (46.2) | 1038 (50.4) | 1328 (64.5) |
| **October** | 2212 | 1014 (45.8) | 1025 (46.3) | 1109 (50.1) | 1412 (63.8) |
| **November** | 2258 | 843 (37.3) | 850 (37.6) | 920 (40.7) | 1319 (58.4) |
| **December** | 2268 | 883 (38.9) | 917 (40.4) | 971 (42.8) | 1237 (54.5) |
| **January** | 2274 | 1307 (57.5) | 1301 (57.2) | 1327 (58.4) | 1254 (55.1) |
| **February** | 2276 | 1598 (70.2) | 1591 (69.9) | 1598 (70.2) | 1097 (48.2) |
| **March** | 2276 | 1584 (69.6) | 1580 (69.4) | 1578 (69.3) | 1152 (50.6) |
| **April** | 2277 | 1485 (65.2) | 1477 (64.9) | 1483 (65.1) | 1146 (50.3) |
