# Supplementary material for: The relationship of within-individual and between-individual variation in mental health with bodyweight: An exploratory longitudinal study
Source: PLoS One. 2024 Jan 10;19(1):e0295117. doi: 10.1371/journal.pone.0295117 (PMC10781195; doi:10.1371/journal.pone.0295117)
Supplement: S3 Table — Estimates of odds ratios with confidence intervals not including 1 are marked in bold. PSS = Perceived Stress Score, PHQ = Patient Health Questionnaire, GAD = Generalised Anxiety Disorder questionnaire. (DOCX) [file pone.0295117.s003.docx]

***Table S3.*** ***Association between baseline characteristics and missingness*** *(i.e., whether the participant had at least one missing data point for the respective outcome at any timepoint) using logistic regression.* *Estimates of odds ratios with confidence intervals not including 1 are marked in bold. PSS = Perceived Stress Score, PHQ = Patient Health Questionnaire, GAD = Generalised Anxiety Disorder questionnaire.*

|  | **Weight** | **Stress (PSS-10)** | **Depressive symptoms (PHQ-8)** | **Anxiety symptoms (GAD-7)** |
| --- | --- | --- | --- | --- |
| **Baseline characteristic** | **Odds Ratio (95% CI)** | | | |
| **Sex (ref: Male)** | **0.81 (0.66 to 0.98)** | 0.99 (0.75 to 1.32) | 1.18 (0.91 to 1.54) | 1.10 (0.85 to 1.42) |
| **BMI (Phase 2 Fenland)** | 1.01 (0.98 to 1.03) | 1.01 (0.98 to 1.05) | 1.01 (0.98 to 1.04) | 1.02 (0.98 to 1.04) |
| **BMI (app study baseline)** | **1.03 (1.01 to 1.05)** | 1.02 (0.99 to 1.05) | 1.01 (0.98 to 1.04) | 1.01 (0.99 to 1.04) |
| **Baseline PHQ score** | **1.05 (1.02 to 1.08)** | 1.01 (0.97 to 1.05) | 1.01 (0.98 to 1.04) | 1.01 (0.97 to 1.04) |
| **Baseline PSS score** | **1.03 (1.01 to 1.04)** | 1.00 (0.98 to 1.02) | 0.99 (0.98 to 1.01) | 1.00 (0.98 to 1.02) |
| **Baseline GAD score** | **1.05 (1.01 to 1.08)** | 1.00 (0.96 to 1.05) | 1.01 (0.97 to 1.05) | 1.02 (0.98 to 1.06) |
| **Age when completed full-time education** | 0.99 (0.97 to 1.01) | 0.99 (0.97 to 1.03) | 1.01 (0.98 to 1.04) | 1.00 (0.97 to 1.02) |
| **Ethnicity (ref: non-White)** | 0.83 (0.42 to 1.73) | 1.04 (0.41 to 3.48) | 0.93 (0.40 to 2.74) | 1.01 (0.43 to 2.95) |
| **Age** | **0.95 (0.93 to 0.96)** | **0.96 (0.94 to 0.98)** | **0.97 (0.95 to 0.99)** | **0.97 (0.95 to 0.98)** |
| **Occupation (ref: Managerial/professional)** |  |  |  |  |
| **Lower managerial/intermediate** | 1.03 (0.79 to 1.35) | 1.08 (0.74 to 1.61) | 1.00 (0.70 to 1.44) | 1.04 (0.74 to 1.49) |
| **Technical/semi-routine** | 1.19 (0.88 to 1.60) | 0.88 (0.60 to 1.32) | 0.84 (0.59 to 1.22) | 0.87 (0.61 to 1.25) |
