# Supplementary material for: The relationship of within-individual and between-individual variation in mental health with bodyweight: An exploratory longitudinal study
Source: PLoS One. 2024 Jan 10;19(1):e0295117. doi: 10.1371/journal.pone.0295117 (PMC10781195; doi:10.1371/journal.pone.0295117)
Supplement: S4 Table — Association between, respectively, stress, depressive symptoms and anxiety symptom scores and log-transformed bodyweight over the study period. All models adjusted for age at baseline, BMI at baseline, sex, education, occupation, restriction level (alternative categorisation), and seasonality (month). Note regression coefficients cannot be interpreted in terms of difference in bodyweight because the outcome is log-transformed. Estimates with confidence intervals not including zero are marked in bold. PSS = Perceived Stress Score, PHQ = Patient Health Questionnaire, GAD = Generalised Anxiety Disorder questionnaire. (DOCX) [file pone.0295117.s004.docx]

***Table S4. Regression models for log-transformed outcomes.*** *Association between, respectively, stress, depressive symptoms and anxiety symptom scores and log-transformed bodyweight over the study period. All models adjusted for age at baseline, BMI at baseline, sex, education, occupation, restriction level (alternative categorisation), and seasonality (month). Note regression coefficients cannot be interpreted in terms of difference in bodyweight because the outcome is log-transformed. Estimates with confidence intervals not including zero are marked in bold. PSS = Perceived Stress Score, PHQ = Patient Health Questionnaire, GAD = Generalised Anxiety Disorder questionnaire.*

|  | **Unstandardised regression coefficient** | **95% confidence interval** |
| --- | --- | --- |
| **Stress (PSS-10)** |  |  |
| Between-individual stress | -0.0000238327 | -0.0006537950 to 0.0006061296 |
| Lagged within-individual stress | -0.0001333356 | -0.0002917488 to 0.0000250776 |
| **Depressive symptoms (PHQ-8)** |  |  |
| Between-individual depression | 0.0007781135 | -0.0004736072 to 0.0020298342 |
| Lagged within-individual depression | **0.0005016077** | **0.0002089656 to 0.0007942498** |
| **Anxiety symptoms (GAD-7)** |  |  |
| Between-individual anxiety | -0.0000834350 | -0.0014496487 to 0.0012827787 |
| Lagged within-individual anxiety | 0.0001353166 | -0.0001513195 to 0.0004219527 |
