# Supplementary material for: The relationship of within-individual and between-individual variation in mental health with bodyweight: An exploratory longitudinal study
Source: PLoS One. 2024 Jan 10;19(1):e0295117. doi: 10.1371/journal.pone.0295117 (PMC10781195; doi:10.1371/journal.pone.0295117)
Supplement: S5 Table — Association of within-individual and between-individual measurements of weight with stress, depressive symptoms and anxiety symptoms over the study period. All models adjusted for age at baseline, BMI at baseline, sex, education, occupation, restriction level, and seasonality (month). Estimate with confidence interval not including zero highlighted in bold. Between-individual measurements = the median of each person’s monthly scores; within-individual measurement = the deviation of each monthly score from that person’s median. PSS = Perceived Stress Score, PHQ = Patient Health Questionnaire, GAD = Generalised Anxiety Disorder questionnaire. (DOCX) [file pone.0295117.s005.docx]

***Table S5.*** ***Regression models for reverse direction of association****. Association of within-individual and between-individual measurements of weight with stress, depressive symptoms and anxiety symptoms over the study period. All models adjusted for age at baseline, BMI at baseline, sex, education, occupation, restriction level, and seasonality (month). Estimate with confidence interval not including zero highlighted in bold. Between-individual measurements = the median of each person’s monthly scores; within-individual measurement = the deviation of each monthly score from that person’s median. PSS = Perceived Stress Score, PHQ = Patient Health Questionnaire, GAD = Generalised Anxiety Disorder questionnaire.*

|  | **Estimated difference in questionnaire score for every 1kg of weight (95% confidence interval)** | | |
| --- | --- | --- | --- |
|  | **Stress (PSS-10)** | **Depressive symptoms (PHQ-8)** | **Anxiety symptoms (GAD-7)** |
| **Between-individual measurement of weight (kg)** | 0.009 (-0.039 to 0.057) | 0.016 (-0.008 to 0.041) | -.00003 (-0.024 to 0.023) |
| **Lagged within-individual measurement of weight (kg)** | -0.005 (-0.073 to 0.064) | 0.027 (-0.009 to 0.064) | 0.001 (-0.036 to 0.038) |
| **Weight change between assessments (kg)** | -0.052 (-0.160 to 0.055) | 0.019 (-0.041 to 0.078) | 0.015 (-0.042 to 0.073) |
