# Supplementary material for: The relationship of within-individual and between-individual variation in mental health with bodyweight: An exploratory longitudinal study
Source: PLoS One. 2024 Jan 10;19(1):e0295117. doi: 10.1371/journal.pone.0295117 (PMC10781195; doi:10.1371/journal.pone.0295117)
Supplement: S6 Table — Relationship between mental health variables and bodyweight by sociodemographic variables and baseline BMI. In addition to the interaction terms, the model includes the following fixed effects: the main effects of the interaction variables, time, season (month as dummy), restriction level, age, sex, baseline BMI. Estimates with confidence intervals not including zero are marked in bold. PSS = Perceived Stress Score, PHQ = Patient Health Questionnaire, GAD = Generalised Anxiety Disorder questionnaire. (DOCX) [file pone.0295117.s006.docx]

***Table S6.*** ***Sub-group analyses****. Relationship between mental health variables and bodyweight by sociodemographic variables and baseline BMI. In addition to the interaction terms, the model includes the following fixed effects: the main effects of the interaction variables, time, season (month as dummy), restriction level, age, sex, baseline BMI. Estimates with confidence intervals not including zero are marked in bold. PSS = Perceived Stress Score, PHQ = Patient Health Questionnaire, GAD = Generalised Anxiety Disorder questionnaire.*

|  | **Unstandardised estimate** | **95% confidence interval** |
| --- | --- | --- |
| **Stress (PSS-10)** |  |  |
| Between-individual stress * age | 0.00 | -0.01 to 0.01 |
| Lagged within-individual stress * age | -0.00 | -0.003 to 0.001 |
| Between-individual stress * sex | -0.05 | -0.15 to 0.05 |
| Lagged within-individual stress * sex | 0.01 | -0.01 to 0.04 |
| Between-individual stress * education | 0.00 | -0.01 to 0.01 |
| Lagged within-individual stress * education | -0.00 | -0.004 to 0.001 |
| Between-individual stress * occupation(Lower managerial/intermediate) | -0.06 | -0.21 to 0.10 |
| Between-individual stress * occupation(Professional/Higher managerial) | 0.01 | -0.12 to 0.15 |
| Lagged within-individual stress * occupation(Lower managerial/intermediate) | -0.02 | -0.06 to 0.03 |
| Lagged within-individual stress * occupation(Professional/Higher managerial) | 0.02 | -0.01 to 0.06 |
| Between-individual stress * baseline BMI | 0.00 | -0.01 to 0.01 |
| Lagged within-individual stress * baseline BMI | 0.00 | -0.002 to 0.003 |
| **Depressive symptoms (PHQ-8)** |  |  |
| Between-individual depression * age | 0.01 | -0.01 to 0.02 |
| Lagged within-individual depression * age | -0.00 | -0.003 to 0.003 |
| Between-individual depression * sex | -0.02 | -0.23 to 0.19 |
| Lagged within-individual depression * sex | 0.02 | -0.03 to 0.07 |
| Between-individual depression * education | 0.02 | -0.00 to 0.04 |
| Lagged within-individual depression * education | -0.00 | -0.01 to 0.00 |
| Between-individual depression * occupation(Lower managerial/intermediate) | -0.11 | -0.4 to 0.18 |
| Between-individual depression * occupation(Professional/Higher managerial) | 0.10 | -0.14 to 0.35 |
| Lagged within-individual depression * occupation(Lower managerial/intermediate) | 0.03 | -0.05 to 0.76 |
| Lagged within-individual depression * occupation(Professional/Higher managerial) | 0.04 | -0.03 to 0.10 |
| Between-individual depression * baseline BMI | 0.01 | -0.01 to 0.02 |
| Lagged within-individual depression * baseline BMI | **0.01** | **0.001 to 0.011** |
| **Anxiety symptoms (GAD-7)** |  |  |
| Between-individual anxiety * age | 0.01 | -0.01 to 0.02 |
| Lagged within-individual anxiety * age | -0.000 | -0.003 to 0.003 |
| Between-individual anxiety * sex | -0.06 | -0.28 to 0.17 |
| Lagged within-individual anxiety * sex | 0.04 | -0.01 to 0.09 |
| Between-individual anxiety * education | 0.02 | -0.01 to 0.04 |
| Lagged within-individual anxiety * education | 0.00 | -0.01 to 0.01 |
| Between-individual anxiety * occupation(Lower managerial/intermediate) | -0.08 | -0.4 to 0.25 |
| Between-individual anxiety * occupation(Professional/Higher managerial) | 0.09 | -0.18 to 0.36 |
| Lagged within-individual anxiety * occupation(Lower managerial/intermediate) | -0.01 | -0.09 to 0.07 |
| Lagged within-individual anxiety * occupation(Professional/Higher managerial) | 0.02 | -0.05 to 0.09 |
| Between-individual anxiety * baseline BMI | 0.00 | -0.02 to 0.02 |
| Lagged within-individual anxiety * baseline BMI | 0.00 | -0.00 to 0.01 |
