# Supplementary material for: The relationship of within-individual and between-individual variation in mental health with bodyweight: An exploratory longitudinal study
Source: PLoS One. 2024 Jan 10;19(1):e0295117. doi: 10.1371/journal.pone.0295117 (PMC10781195; doi:10.1371/journal.pone.0295117)
Supplement: Attachment — Submitted filename: Response to Reviewers.docx [file pone.0295117.s009.docx]

# Reviewer 1

## Comment 1

Reviewer #1: I thought the authors did a very comprehensive job of addressing my comments and thank them for being open to some alternative suggestions/ideas.

I suggest one very minor final change. I think the title should be called 'an exploratory longitudinal study' given that the authors describe the study as such in the manuscript and the p value isn't correct for multiple comparisons. Assuming the authors are happy to do this then I don't need/want to see this again, and would prefer the editor signs off on my behalf once the revision comes in.

Well done on this work.

## Response 1

Thank you very much, we really appreciate the reviewers taking the time to review and help improve our manuscript. Thank you for the suggestion, we updated the title as suggested:

“The relationship of within-individual and between-individual variation in mental health with bodyweight: An exploratory longitudinal study”

# Reviewer 2

## Comment 1

Reviewer #2: Dear authors,

Thank you very much for implementing my and the other reviewers’ suggestions. I think, it reads much clearer now.
There are just very minor suggestions to further improve clarity:

## Response 1

Thank you very much, we really appreciate you taking the time to review and help improve our manuscript.

## Comment 2

Results:
ll. 169-172: In the methods section, it still sounds like you excluded the whole participant if BMI or weight loss were implausible. Later in the results section (ll. 271-273) and your response to my comment, it sounds more like only the datapoint of BMI or weight loss was removed, while the rest of the participant’s data were still used.

## Response 2

Thank you for spotting this. We have amended the text to clarify that we excluded only specific data points rather than the entire participant:

“For the present analysis, we excluded weight entries that indicated a biologically implausible BMI of <12 or >70kg/m2 [26,27] or biologically implausible weight loss between two timepoints as defined by Chen et al. [28].” (p. 8, line 173)

Comment 3

ll. 276-278: You added the average number of values for each mental health outcome, which is a good idea, I think. It would be good to have an indication of how long the assessment period was and therefore, how many datapoints they could maximally enter. In your methods section, you give the month/year when participants can start download the app and enter datapoints to it, and when the last month/year is for data entry. To make it easier for the reader, it would be helpful if you could calculate the number of months and add this information here to the results section. This would help the reader to bring the average number of values into context and to determine if people entered many or only few data points.

Response 3

Thank you for pointing this out. We have now added a sentence to the methods section to clarify:

“Those in the app sub-study were able to download the app from 6th August 2020, and the study finished on 30th April 2021 (when the app also closed to further measurement entry). The number of monthly measurements participants could provide depended on the timepoint when they entered the study (e.g., a participant entering in August 2020 could add a total of 9 measurements up until April 2021, a participant entering in September could add 8, etc.)”. (p. 7, lines 157-160)

Comment 4

Table 1: You have “a” described under your table, but “4” as a footnote. As both describes variables in the table, also the information about SES could be under the table as “b”.

Response 4

We have now changed it so that it’s consistent (using “a” and “b” to add notes at the bottom of the table to describe variables in more detail). (Table 1, pp. 13-14)

Comment 5

Figure 1: Sorry for being pedantic, for graph b, the label “time” for the x-axis is missing. As you mentioned in your answer to reviewer 1, please do work with the editors to get a higher quality figure. It is really hard to read this figure.

## Response 5

Thank you for spotting this. We have edited the figure so that the x-axis labelling is now visible. Also, apologies that the figure is coming out blurry. It is unclear why it is appearing so blurry in the pdf for peer review. It looks very crisp on my screen, and the file has a resolution of 300dpi, which should be sufficient. If it turns up blurry during copy editing, we will make sure to address this.
